# Cost-Effectiveness of Nivolumab Plus Chemotherapy vs. Chemotherapy as First-Line Treatment for Advanced Gastric Cancer/Gastroesophageal Junction Cancer/Esophagel Adenocarcinoma in China

**DOI:** 10.3389/fonc.2022.851522

**Published:** 2022-04-20

**Authors:** Yamin Shu, Yufeng Ding, Qilin Zhang

**Affiliations:** ^1^ Department of Pharmacy, Tongji Hospital, Tongji Medical College, Huazhong University of Science and Technology, Wuhan, China; ^2^ Department of Pharmacy, Union Hospital, Tongji Medical College, Huazhong University of Science and Technology, Wuhan, China

**Keywords:** cost-effectiveness analysis, gastric cancer/gastroesophageal junction cancer/esophageal adenocarcinoma, nivolumab, CheckMate 649 trial, first-line treatment

## Abstract

**Objective:**

The purpose of this study was to evaluate the cost-effectiveness of nivolumab plus chemotherapy vs. chemotherapy as first-line therapy in patients with advanced gastric cancer/gastroesophageal junction cancer/esophageal adenocarcinoma from the perspective of the Chinese healthcare system.

**Methods:**

This economic evaluation used a state-transition Markov model to assess the cost and effectiveness of nivolumab plus chemotherapy vs. chemotherapy as first-line treatment for advanced gastric cancer/gastroesophageal junction cancer/esophageal adenocarcinoma. The characteristics of patients in the model came from a phase 3 open-label randomized clinical trial (CheckMate 649). Key clinical data were based on the CheckMate 649 trial conducted from March 2017 to April 2019, and costs and utilities were collected from the published literature. The total cost of treatment per patient, quality-adjusted life-years (QALYs), and incremental cost-effectiveness ratio (ICER) were calculated for the two treatment strategies. Deterministic sensitivity analysis and probabilistic sensitivity analysis were performed.

**Results:**

In the baseline analysis, the incremental effectiveness and cost of nivolumab plus chemotherapy vs. chemotherapy were 0.28 QALYs and $78,626.53, resulting in an ICER of $278,658.71/QALY, higher than the willingness-to-pay (WTP) threshold of China ($31,498.70/QALY). The model was sensitive to the duration of progression-free survival (PFS) for the nivolumab plus chemotherapy group, the cost of nivolumab per 100 mg, and the utility of PFS.

**Conclusion:**

Nivolumab plus chemotherapy was clearly not a cost-effective treatment strategy compared with chemotherapy as first-line therapy for patients with advanced gastric cancer/gastroesophageal junction cancer/esophageal adenocarcinoma in China. Reducing the price of nivolumab may improve its cost-effectiveness.

## Introduction

Gastric cancer (GC), including gastroesophageal junction cancer (GEJC), is the fifth most common cancer and is the fourth leading cause of cancer mortality worldwide (1, 2). In China, the morbidity and mortality of GC rank second among malignant tumors. Approximately 80% of patients diagnosed with GC are advanced metastatic disease, which have a very poor prognosis, with a 5-year survival rate of only about 5% (3, 4). Fluoropyrimidine plus platinum-based chemotherapy remains the standard first-line therapy for patients with non-operative radical or human epidermal growth factor 2 (HER2)-negative advanced or metastatic GC/GEJC by the National Comprehensive Cancer Network (NCCN), the European Society for Medical Oncology (ESMO), and the Chinese Society of Clinical Oncology (CSCO) (5–7), despite poor efficacy. For HER2-positive GC/GEJC, a targeted agent such as trastuzumab is recommended as first-line therapy, but the known incidence of HER2-positive in GC/GEJC was only about 20% (8, 9). The majority of patients with advanced GC/GEJC still lack innovative treatment options.

Nivolumab, a fully human IgG4 monoclonal antibody, can block the binding of programmed death-1 (PD-1) with its ligand PD-L1 and restore the function of T cell activation and cytokine production, thus achieving excellent antitumor effects. It has been proved to prominently provide improved survival benefits and quality of life for patients with non-small cell lung cancer (NSCLC), renal cancer, head and neck cancer, melanoma, and other cancers who previously had few treatment options (10–13). PD-L1 expression on tumor cells and tumor-associated immune cells (combined positive score [CPS]) showed better efficacy than PD-L1 expression on tumor cells of immune checkpoint inhibitors in the treatment of advanced GC/GEJC/esophageal adenocarcinoma (9).

The world’s first global multicenter, randomized, open-label, phase 3 clinical study of the first-line immunotherapy combined with chemotherapy for patients with previously untreated, unresectable advanced, or metastatic GC/GEJC/esophageal adenocarcinoma is the CheckMate 649 trial, which is designed to evaluate the efficacy of nivolumab plus chemotherapy compared with chemotherapy alone (9). Results were published in July 2021 and demonstrated that nivolumab plus chemotherapy resulted in significant improvements in overall survival (OS) (14.4 vs. 11.1 months, hazard ratio (HR) = 0.71, 98.4% CI, 0.59–0.86, *p* < 0.0001) and progression-free survival (PFS) (7.7 vs. 6.05 months, HR = 0.68, 98% CI, 0.56–0.81, *p* < 0.0001) when compared with chemotherapy alone in PD-L1 CPS ≥5 GC/GEJC/esophageal adenocarcinoma patients (9).

Based on the CheckMate 649 study, on April 16, 2021, the Food and Drug Administration (FDA) approved nivolumab in combination with fluorouracil and platinum agents as the new first-line treatment strategy for patients with advanced or metastatic GC/GEJC/esophageal adenocarcinoma, regardless of PD-L1 expression status, followed by NCCN Guidelines (2021 edition) recommended. Just over 4 months later, on August 31, 2021, the National Medical Products Administration (NMPA) approved the same indication in China. In the CheckMate 649 trial, the Chinese population showed a trend of greater benefit as compared with the global population, for example, 39% vs. 20% reduction in the risk of death and 43% vs. 23% reduction in the risk of disease progression or death. The first-line treatment of HER2-negative advanced GC patients in China has been facing a huge gap in innovative treatment for a long time. The emergence of nivolumab has brought an unprecedented breakthrough in this field. Therefore, nivolumab immunotherapy combined with chemotherapy has been recommended as the first-line therapy for HER2-negative advanced or metastatic GC with PD-L1 CPS ≥5 in the latest CSCO (2021 edition) guidelines.

Despite the longer survival benefit of nivolumab, its high cost also increases the economic burden on patients’ families and society. The cost-effectiveness of first-line treatment of advanced GC/GEJC with nivolumab plus chemotherapy has not, to our knowledge, been evaluated in China and other countries. The primary objective of this study was to estimate the cost-effectiveness of nivolumab plus chemotherapy compared with chemotherapy alone as first-line treatment for advanced or metastatic PD-L1 CPS ≥5 GC/GEJC/esophageal adenocarcinoma patients from the perspective of the Chinese healthcare system.

## Methods

### Model Structure

**Figure 1:**
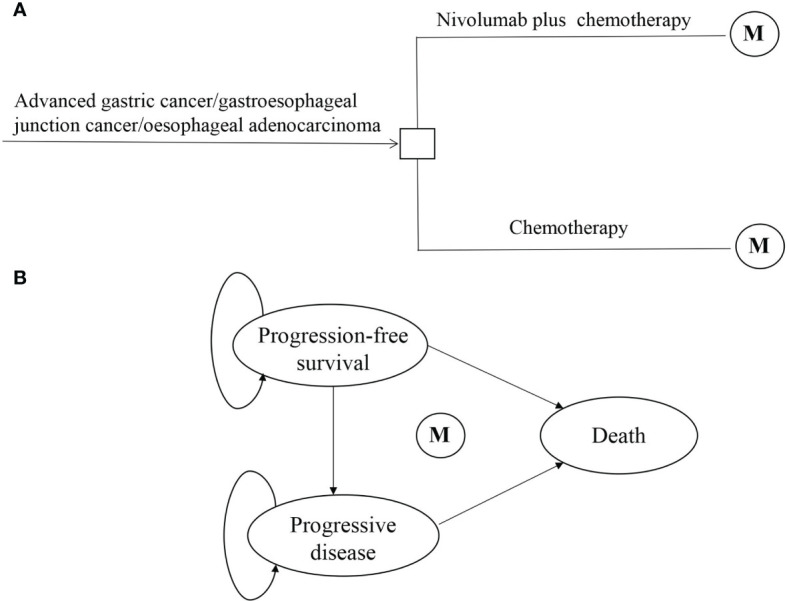
Model structure of a decision tree combining the Markov state transition model with the 3 health states. **(A)** Decision tree. **(B)** Markov state transition model. M, Markov node.

This economic evaluation used a state-transition Markov model to estimate the cost and effectiveness associated with nivolumab plus chemotherapy vs. chemotherapy as first-line treatment for advanced GC/GEJC/esophageal adenocarcinoma in China (**Figure 1**). Patients were simulated through three mutually exclusive health states: PFS, progressive disease (PD), and death. All began in PFS with advanced disease, and patients either remained in their assigned health state or progressed to a new health state during each Markov cycle. It was assumed that all patients received first-line treatment until disease progression, and both groups could receive second-line treatment until death.

The time horizon of the model simulation was 5 years, and each Markov cycle represented 1 month in the model. The primary endpoints of the model were the total cost of treatment per patient, quality-adjusted life-years (QALYs), and incremental cost-effectiveness ratio (ICER). The formula used to calculate the ICER is as follows: ICER = [Cost (nivolumab plus chemotherapy) − Cost (chemotherapy)]/[QALY (nivolumab plus chemotherapy) − QALY (chemotherapy)]. The future costs and survival estimates were adjusted at a discount rate of 3% per year according to the WHO guidelines for pharmacoeconomic evaluations (14). ICER was compared with a willingness-to-pay (WTP) threshold of 3× the per-capita gross domestic product (GDP) of China in 2020 ($31,498.70). All costs had been adjusted to 2020 prices according to the local Consumer Price Index and were presented in US dollars ($1 = ¥6.9). The Markov model was performed in TreeAge Pro 2019 software (Williamstown, MA, USA), and statistical analyses were performed in R software (version 4.0.5, Vienna, Austria). This economic analysis was based on a published randomized clinical trial, and a mathematical model was used. Thus, the study did not require approval from an institutional review board or ethics committee.

### Clinical Data and Transition Probabilities

The survival benefits and safety data of nivolumab plus chemotherapy and chemotherapy were based on the results of the CheckMate 649 trial (ClinicalTrials.gov number, NCT02872116), a multicenter, randomized, open-label, phase 3 trial (9). Eligible patients conformed to the following conditions: 1) 18 years of age or older, with previously untreated, unresectable advanced, or metastatic GC/GEJC/esophageal adenocarcinoma, regardless of PD-L1 expression; 2) measurable (at least one lesion) or evaluable disease per Response Evaluation Criteria in Solid Tumors (RECIST), version 1.1, and Eastern Cooperative Oncology Group performance status of 0 or 1; 3) adequate organ function and availability to provide a fresh or archival tumor sample to evaluate PD-L1; and 4) patients with prior adjuvant or neoadjuvant chemotherapy, radiotherapy, and/or chemoradiotherapy (administered at least 6 months before randomization) were allowed. Patients were randomly assigned in a 1:1 ratio to nivolumab plus chemotherapy (PD-L1 CPS ≥5, n = 473) or chemotherapy alone (PD-L1 CPS ≥5, n = 482). Patients were administered nivolumab (360 mg every 3 weeks or 240 mg every 2 weeks) plus chemotherapy (XELOX [capecitabine 1,000 mg/m^2^ twice daily, days 1 to 14 and oxaliplatin 130 mg/m^2^, day 1, every 3 weeks] or FOLFOX [leucovorin 400 mg/m^2^, day 1; fluorouracil 400 mg/m^2^, day 1 and 1200 mg/m^2^, days 1 and 2; and oxaliplatin 85 mg/m^2^, day 1, every 2 weeks]) or chemotherapy alone. Treatment continued until disease progression or unacceptable toxicity. The median OS was 14.4 months (95% CI, 13.1–16.2) in the nivolumab plus chemotherapy group and 11.1 months (95% CI, 10.0–12.1) in the chemotherapy group. The median PFS was 7.7 months (95% CI, 7.0–9.2) in the nivolumab plus chemotherapy group and 6.05 months (95% CI, 5.6–6.9) in the chemotherapy group.

**Figure 2:**
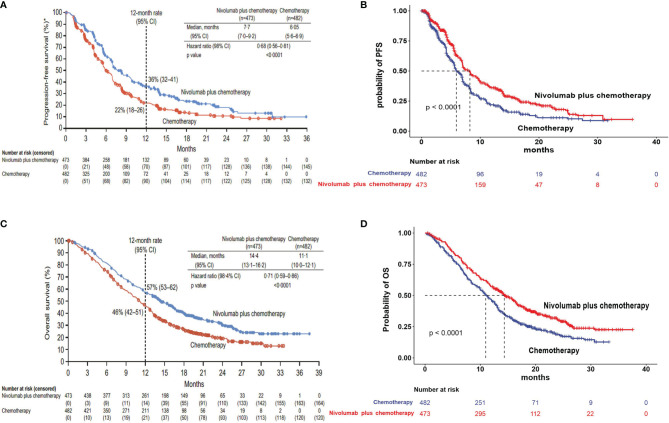
Model estimated PFS and OS were plotted, together with the original Kaplan–Meier PFS and OS curves from the CheckMate 649 trial, respectively. **(A)** Kaplan–Meier curve of the progression-free survival from the CheckMate 649 trial. **(B)** Simulate progression-free survival curve for the nivolumab plus chemotherapy and chemotherapy. **(C)** Kaplan–Meier curve of overall survival from the CheckMate 649 trial. **(D)** Simulate overall survival curve for the nivolumab plus chemotherapy and chemotherapy. PFS, progression-free survival; OS, overall survival.

The Kaplan–Meier survival curves from the CheckMate 649 trial were used to estimate transition probabilities between different health states. First, OS and PFS data points were extracted from the corresponding Kaplan–Meier survival curves using the GetData Graph Digitizer software (version 2.26), which digitized data points from an image file. Second, virtual data comprised follow-up time and the same initial number at risk, which closely reproduced the digitized Kaplan–Meier curves, and R software was used to reconstruct the Kaplan–Meier curve of the obtained data (**Figure 2**). Third, to predict survival beyond the observation period, the proportions of patients with PFS and OS were calculated by using the Weibull distribution. Finally, the Weibull distribution parameters, scale (λ) and shape (γ) parameters, SE, and 95% CI were computed using R (**Table 1**). Formula S(t) = exp(−λt^γ^) was used to calculate the survival probability at time t, and the transition probabilities between different health states at a given cycle t were estimated by formula P(t) = 1 − exp[λ(t − 1)^γ^ − λt^γ^] (15, 16). The background mortality rate from PFS to death state was derived from the natural death rate of the Chinese population in 2020 (0.707%) (17).

**Table 1:** Weibull parameters of model estimated for progression-free and overall survival curves.

Group		Parameter	Mean	SE	95% CI	
					Low	Up
Nivolumab plus chemotherapy	PFS	Scale (λ) Shape (γ)	0.047288 1.186735	0.006835 0.051729	0.035621 1.089558	0.062776 1.292580
	OS	Scale (λ) Shape (γ)	0.021699 1.267705	0.004037 0.061001	0.015069 1.153611	0.031245 1.393084
Chemotherapy	PFS	Scale (λ) Shape (γ)	0.073625 1.170952	0.009085 0.047834	0.057809 1.080855	0.093768 1.268560
	OS	Scale (λ) Shape (γ)	0.031801 1.267607	0.005104 0.055288	0.023217 1.163747	0.043558 1.380736

PFS, progression-free survival; OS, overall survival.

### Costs and Utilities

Costs were estimated from the perspective of the Chinese healthcare system. The direct medical cost components, that is, the costs of first-line and subsequent treatment, management of treatment-related grade 3–4 serious adverse events (SAEs), laboratory tests and radiological examinations, best supportive care (BSC), cost of salvage therapy per cycle, routine follow-up, and terminal care in end of life, were included in the model (**Table 2**). In calculating dosage amounts, a body weight of 65 kg and a height of 1.64 m were used, resulting in a body surface area of 1.72 m^2^ for typical patients (24). In addition, to better reflect the cost of first-line treatment in real-world settings, the duration of these treatments was adjusted based on the median treatment cycles reported in the CheckMate 649 trial. Only grade 3 or higher SAEs with an incidence of >5% at least in one group were incorporated into the model, including anemia, decreased neutrophil count, neutropenia, and increased lipase. The costs related to SAEs were calculated by multiplying the incidence of the SAEs by the costs of managing the SAEs per event. After disease progression, patients could subsequently receive salvage chemotherapy and supportive care. All costs were derived from local hospitals or previously published literature (18–22).

**Table 2:** Model economic parameters and the range of the sensitivity analysis.

Variables	Base case (range)	Distribution	Source
Costs ($)			
Nivolumab (100 mg)	1,342.03 (1,073.62–1,610.44)	Triangle	Local charge
Oxaliplatin (100 mg)	90.00 (72.00–108.00)	Triangle	Local charge
Capecitabine (1,000 mg)	6.38 (5.10–7.66)	Triangle	Local charge
Leucovorin (100 mg)	2.22 (1.78–2.66)	Triangle	Local charge
Fluorouracil (1,000 mg)	26.67 (21.34–32.00)	Triangle	Local charge
Cost of salvage therapy per cycle	478.82 (383.06–574.58)	Triangle	Local charge
Routine follow-up cost per cycle	80.71 (64.57–96.85)	Triangle	(18)
Cost of tests and radiological examinations per cycle	141.29 (113.03–169.55)	Triangle	(19)
Cost of supportive care per cycle	164.57 (131.66–197.48)	Triangle	(18)
Cost of terminal care in end of life	1,460.30 (1,168.24–1,752.36)	Triangle	(18)
Costs of serious adverse events			
Anemia	508.20 (381.2–635.3)	Triangle	(20)
Neutrophil count decreased	534.40 (427.52–641.28)	Triangle	(21)
Neutropenia	466.00 (372.80–559.20)	Triangle	(20)
Lipase increased	44.30 (35.44–53.16)	Triangle	(22)
Risks of serious adverse events in nivolumab plus chemotherapy group (grade 3 or 4) %			
Anemia	6.46 (5.17–7.75)	Beta	(9)
Neutrophil count decreased	11.40 (9.12–13.68)	Beta	(9)
Neutropenia	16.21 (12.97–19.45)	Beta	(9)
Lipase increased	6.18 (4.94–7.42)	Beta	(9)
Risks of serious adverse events in chemotherapy group (grade 3 or 4) %			
Anemia	2.74 (2.19–3.29)	Beta	(9)
Neutrophil count decreased	8.74 (6.99–10.49)	Beta	(9)
Neutropenia	12.13 (9.70–14.56)	Beta	(9)
Lipase increased	2.09 (1.67–2.51)	Beta	(9)
Utility value			
PFS	0.797 (0.638–0.956)	Beta	(23)
PD	0.577 (0.462–0.692)	Beta	(23)
Body surface area (m^2^)	1.72 (1.38–2.06)	Triangle	(24)
Discount rate (%)	3 (0–8)	Fixed in PSA	(14)

PFS, progression-free survival; PD, progressive disease; PSA, probabilistic sensitivity analyses.

The CheckMate 649 trial had evaluated the health-related quality of life (HRQoL), but it has not been published. The baseline utility estimates for PFS and PD health states were derived from previously published literature (23), with 0 indicating death and 1 indicating perfect health. To simplify the model, the disutility of SAEs in the model was not considered, as the effect of SAEs was assumed to be captured in the utility values. Furthermore, a half-cycle correction was implemented to the outcomes, according to the TreeAge Pro 2019 manual and Guidelines for pharmacoeconomic evaluation in China.

### Sensitivity Analyses

To assess the robustness of the model and the uncertainty in parameter estimation, deterministic sensitivity analysis and probabilistic sensitivity analysis were performed in this research. In the deterministic sensitivity analysis, relevant variables were tested one by one at the upper and lower limits of plausible ranges, to explore the impact of each parameter on ICER. The result of the deterministic sensitivity analysis is presented in a tornado diagram. To determine the effect of variation in multiple parameters simultaneously, a probabilistic sensitivity analysis with 1,000 Monte Carlo simulations was performed, in which the parameters were changed with a specific pattern of distribution. A cost-effectiveness acceptability curve and probabilistic scatter plot were given to show the probability of the cost-effectiveness simulations at various WTP thresholds.

To investigate the uncertainty of economic outcomes caused by the differences in race, exploratory subgroup analyses were performed for the prespecified subgroup that was reported in PD-L1 CPS ≥5 GC/GEJC/esophageal adenocarcinoma patients in the CheckMate 649 trial by varying the HR for OS.

## Results

### Incremental Cost-Effectiveness Ratios

The primary analysis results of the model are listed in **Table 3**. In the base case, first-line treatment with nivolumab plus chemotherapy resulted in a cost of $88,190.33 and survival of 1.11 QALYs per patient. Treatment with chemotherapy resulted in a cost of $9,563.80 and survival of 0.82 QALYs. Nivolumab plus chemotherapy provided an additional $78,626.53 and conferred an additional 0.28 QALYs, leading to an ICER of $278,658.71/QALY. At the Chinese cost-effectiveness WTP threshold of $31,498.70/QALY, nivolumab plus chemotherapy was clearly not a cost-effective treatment strategy compared with chemotherapy.

**Table 3:** The cost and outcome results of the cost-effectiveness analysis.

Parameters	Nivolumab plus chemotherapy	Chemotherapy
Costs ($)		
PFS state	83,110.53	5,183.48
PD state	5,079.79	4,380.32
Total cost	88,190.33	9,563.80
Incremental costs ($)	78,626.53	/
Effectiveness (QALYs)		
PFS state	0.82	0.59
PD state	0.28	0.23
Total effectiveness	1.11	0.82
Incremental effectiveness (QALYs)	0.28	/
ICER ($/QALY)	278,658.71	/

PFS, progression-free survival; PD, progressive disease; QALYs, Quality-adjusted life-years; ICER, incremental cost-effectiveness ratios.

### Sensitivity Analyses

**Figure 3:**
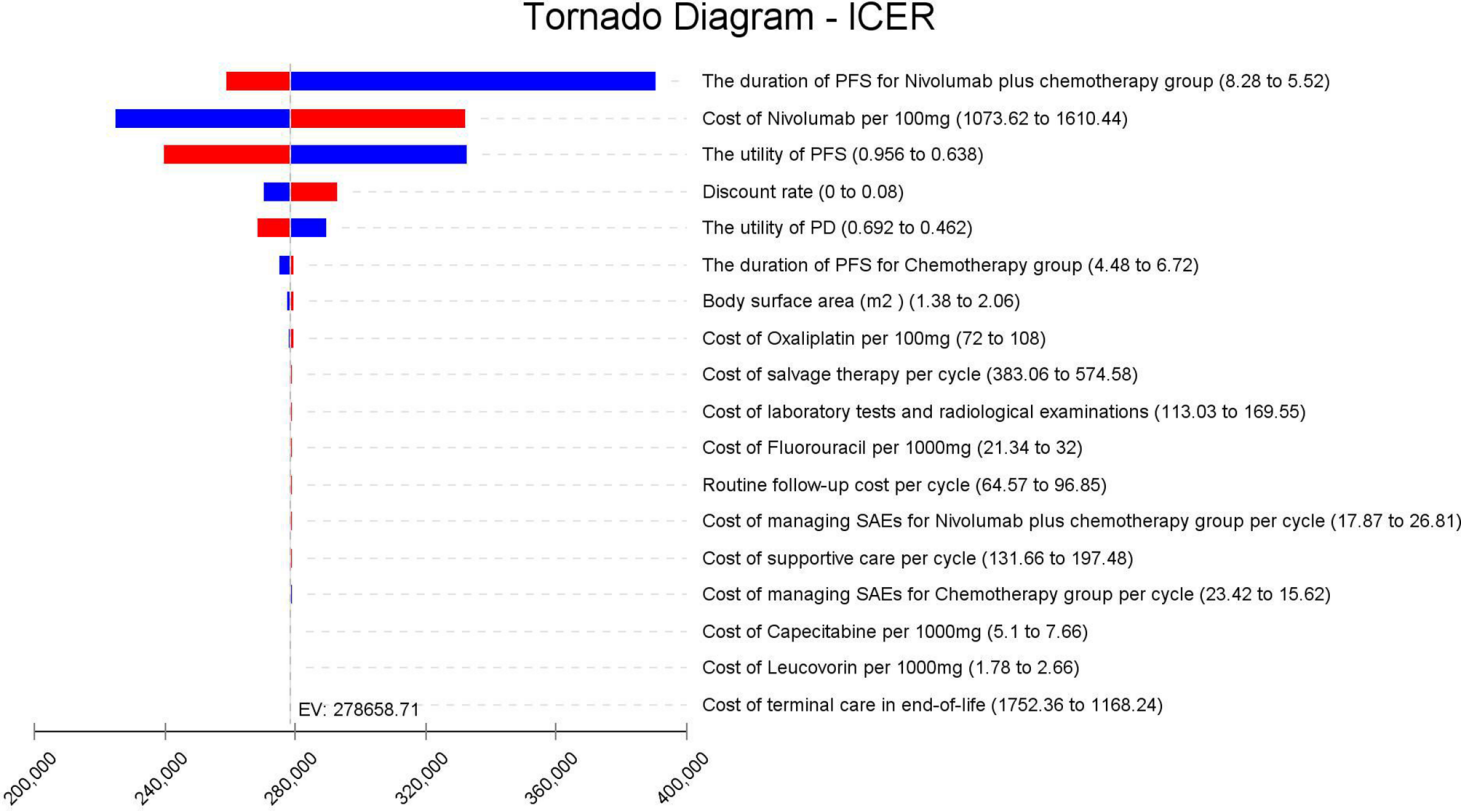
Tornado diagram of one-way sensitivity analysis. It summarizes the results of one-way sensitivity analysis, which lists influential parameters in descending order according to their effect on the ICER over the variation of each parameter value. ICER, incremental cost-effectiveness ratios; PFS, progression-free survival; PD, progressive disease; SAEs, serious adverse events.

**Figure 4:**
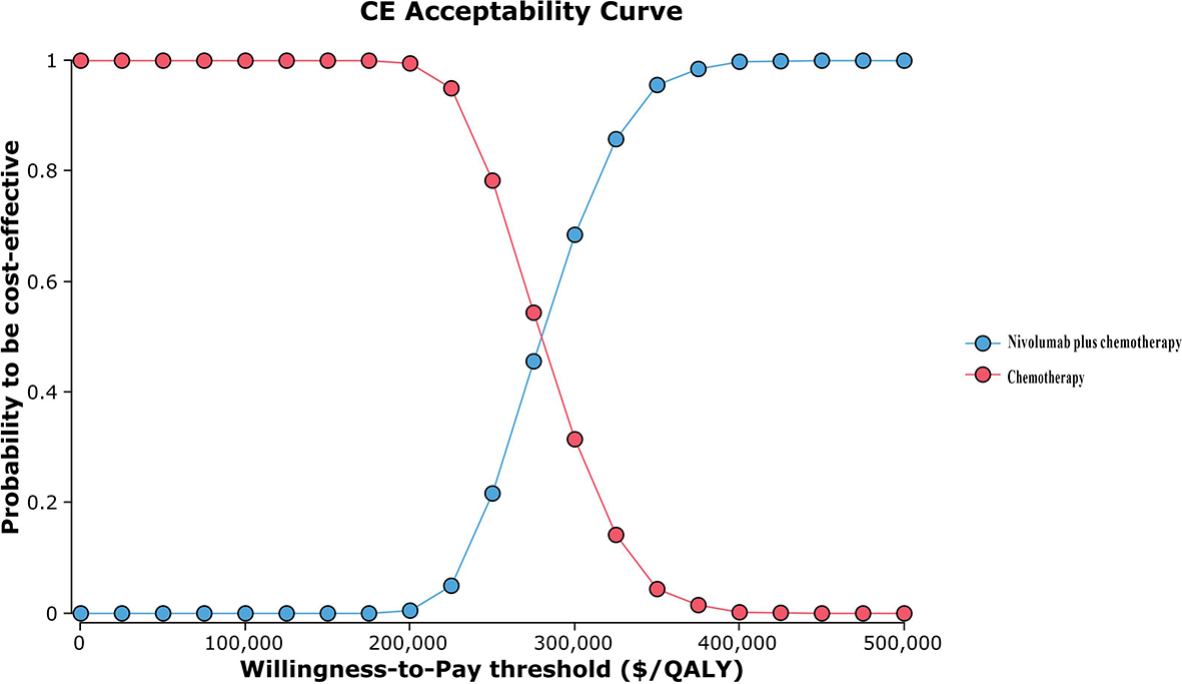
Cost-effectiveness acceptability curve. QALY, quality-adjusted life-year; CE, cost-effectiveness.

**Figure 5:**
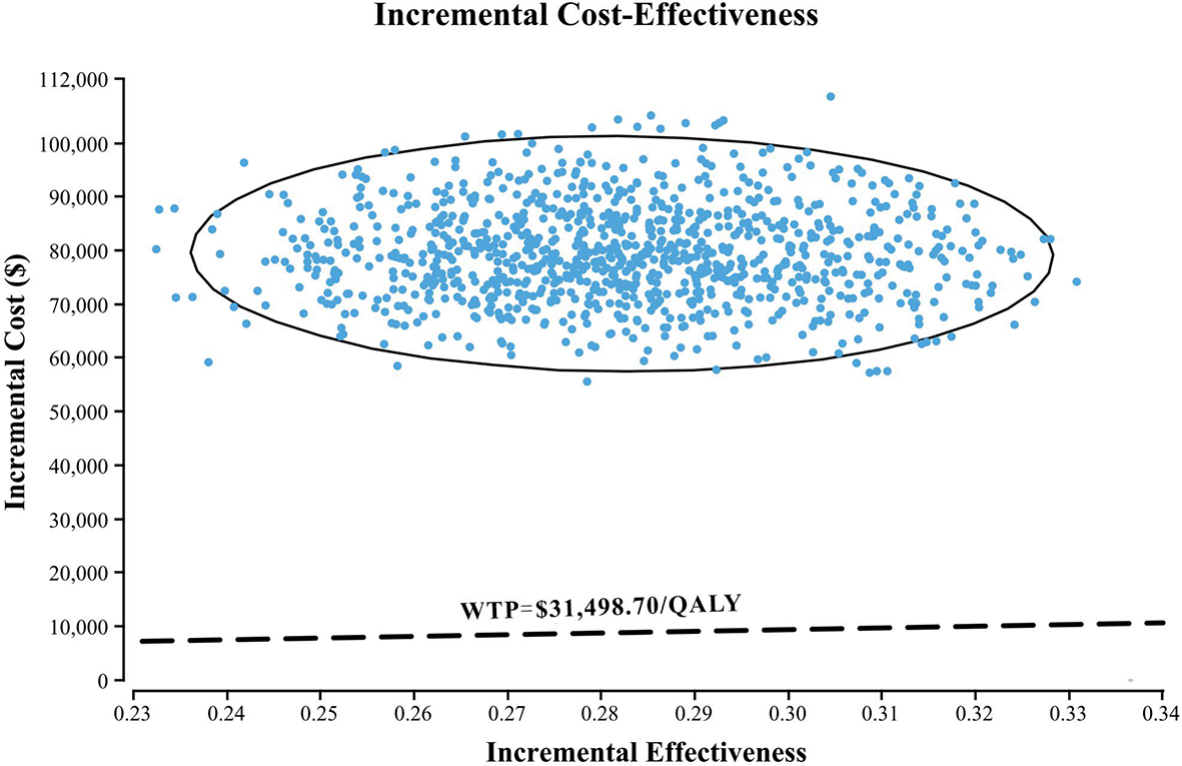
A probabilistic scatter plot of the ICER between the nivolumab plus chemotherapy and chemotherapy. Each dot represents the ICER for 1 simulation. An ellipse means 95% CI. Dots that are located below the ICER threshold represent cost-effective simulations. WTP, willingness to pay; QALY, quality-adjusted life-year; ICER, incremental cost-effectiveness ratios.

The results of one-way sensitivity analyses are shown in a tornado diagram (**Figure 3**). The variables that had the greatest influence on the ICER were the duration of PFS for the nivolumab plus chemotherapy group, the cost of nivolumab per 100 mg, and the utility of PFS. Other parameters such as discount rate, body surface area (m^2^), and costs of SAEs had a moderate or mild impact on ICER. However, any of the tested variables’ upper or lower limits were unable to change the cost-effective treatment strategy from chemotherapy to nivolumab plus chemotherapy, with the ICERs below the thresholds. The probabilistic sensitivity analysis of the primary analysis revealed that the probability of nivolumab plus chemotherapy being cost-effective was 0% at the WTP threshold of $31,498.70/QALY (**Figures 4**, **5**). Treatment with nivolumab plus chemotherapy had a 50% probability to be cost-effective at a WTP threshold of approximately $280,000/QALY, and this probability increased with the rising WTP thresholds (**Figure 4**). According to the sensitivity analyses, the results of the model were very robust. Although our study was based on the subgroup of PD-L1 CPS ≥5, due to the small difference in survival benefit of each subgroup, the results could be generalized to other subgroups regardless of the PD-L1 CPS expression level in the CheckMate 649 trial. However, in comparison with chemotherapy, nivolumab plus chemotherapy was associated with an ICER of $240,678.12/QALY in the Asian population subgroup of PD-L1 CPS ≥5, which was lower than the overall population.

## Discussion

The average early diagnosis rate of GC in China is only about 10%, resulting in a large proportion of patients with advanced GC. The prognosis of advanced GC is relatively poor, and the curative effect is not ideal. CheckMate 649 is the only study to date in the treatment of advanced GC to confirm the dual benefit of PFS and OS achieved by immunotherapy combined with chemotherapy. So far, CheckMate 649 was the largest randomized, global multicenter phase 3 study, which enrolled 2,032 patients at 176 centers in the first-line treatment of advanced GC/GEJC/esophageal adenocarcinoma based on immune checkpoint inhibitors and was published in *Lancet* (9). With 208 participants, China has the highest percentage (13.4%) of patients among all countries; hence, results can be extrapolated to the Chinese population to a large extent.

Nivolumab is the first and currently the only PD-1 inhibitor approved for first-line therapy of advanced GC in China. With the widespread use of nivolumab, the substantial increase in financial burden has become an important concern for decision makers. An economic evaluation of nivolumab has become an urgent need. To our knowledge, this study is the first to evaluate the cost-effectiveness of nivolumab plus chemotherapy as first-line treatment for advanced PD-L1 CPS ≥5 GC/GEJC/esophageal adenocarcinoma patients as recommended by the latest clinical guidelines (7), and our results are of great significance in both China and other countries.

On the basis of the simulated survival model, our analysis showed that nivolumab plus chemotherapy demonstrated an average of 1.11 QALYs, while chemotherapy demonstrated 0.82 QALYs. Nivolumab plus chemotherapy was more effective than chemotherapy by 0.28 QALYs. Furthermore, nivolumab plus chemotherapy was also more expensive with the cost of $88,190.33 compared to $9,563.80 for chemotherapy (+$78,626.53), resulting in an ICER of $278,658.71/QALY, much higher than the WTP value ($31,498.70/QALY) in China. In our study, the subgroup with PD-L1 CPS ≥5 was selected for analysis, because the PFS and OS of this subgroup had the highest survival benefits. At the same cost, since the subgroup with PD-L1 CPS ≥5 was not economical, the other groups were even less economical. In summary, it means that regardless of PD-L1 CPS expression level, nivolumab plus chemotherapy regimen as first-line treatment of advanced GC/GEJC/esophageal adenocarcinoma is not cost-effective in China, despite having a greater survival benefit as evaluated by QALYs.

In the one-way sensitivity analysis, the duration of PFS for the nivolumab plus chemotherapy group, the cost of nivolumab per 100 mg, and the utility of PFS were the most sensitive parameters, which had the greatest influence on the model results. However, within the variation range of each parameter, the ICER value was always higher than the WTP value, which had no influence on the final outcomes, proving the stability of the model. The cost of nivolumab was much higher than the placebo, which was the main reason why it was not cost-effective. We obtained an economical price of nivolumab by changing the price of nivolumab so that ICER was close to or equal to the WTP ($31,498.70/QALY) in China. According to the one-way sensitivity analysis (**Figure 3**), when the price of nivolumab is reduced by 90%, with an ICER of $30,843.63/QALY, it is lower than the WTP in China, and it becomes cost-effective, further supporting the view that nivolumab is currently costly for its clinical value. However, in the Asian population subgroup of PD-L1 CPS ≥5, it becomes cost-effective in China when the price of nivolumab is reduced by 83% (ICER = $31,016.44/QALY). But it does not mean the nivolumab plus chemotherapy in Asians was more cost-effective than in non-Asians because the WTP was varied in different countries. Probability sensitivity analysis showed that the probability of nivolumab plus chemotherapy being cost-effective relative to chemotherapy (ICER below $31,498.70/QALY) was 0%. Nivolumab plus chemotherapy would only be more cost-effective than chemotherapy if WTP exceeded approximately $280,000/QALY. It should be noted that the per-capita GDP of different regions in China varies greatly, among which the WTP of economically developed cities and provinces, such as Beijing, Shanghai, Jiangsu, Fujian, and Zhejiang, are $72,886.96/QALY, $69,297.83/QALY, $55341.30/QALY, $48,046.09/QALY, and $48,021.74/QALY, respectively. However, the nivolumab plus chemotherapy regimen is still not cost-effective in these areas. Additionally, ICER in the nivolumab plus chemotherapy group was higher than the threshold recommended by wealthier developed countries, such as £20,000–30,000 per QALY proposed by the National Institute for Health and Care Excellence (NICE) in the United Kingdom and $150,000 per QALY in the United States (25, 26). It suggests that nivolumab plus chemotherapy may also not be cost-effective as first-line therapy for patients with advanced GC/GEJC/esophageal adenocarcinoma in other developed countries.

At present, most of the pharmacoeconomic studies on advanced GC focused on screening, surgical techniques, and chemoradiation, and few studies focused on immunotherapy (27–30). Moreover, economic studies of nivolumab have also been limited to advanced renal cell carcinoma, NSCLC, and melanoma (31–33). Only one Japanese study showed that the QALYs and expected costs per patient were 0.5295 and JPY 5,018,148 ($45,620) for nivolumab and 0.4379 and JPY 2,054,625 ($18,678) for trifluridine/tipiracil, respectively, for patients with heavily pretreated metastatic GC (34). The ICER of nivolumab vs. trifluridine/tipiracil was JPY 32,352,489 ($294,113) per QALY gained, much higher than the WTP of Japan. Accordingly, nivolumab is not cost-effective compared to trifluridine/tipiracil.

In fact, due to the limited survival, small incremental effect, and high incremental cost of patients with advanced GC, many antitumor drugs are not considered economical. Shiroiwa (23) reported that trastuzumab plus chemotherapy for HER2-positive advanced GC was not cost-effective based on the ToGA trial. Chen et al. (35) conducted a cost-effectiveness analysis of apatinib in patients with advanced GC in China and found that apatinib was not cost-effective with an ICER of $90,154/QALY (WTP = $23,700/QALY). Pharmacoeconomic studies in both China and other countries have found that ramucirumab alone or in combination with paclitaxel does not have a cost-effectiveness advantage in second-line therapy for advanced GC/GEJC (4, 36, 37). Another research demonstrated that among six possible second-line treatment options for patients with advanced GC who have failed previous chemotherapy—irinotecan, docetaxel, paclitaxel, ramucirumab, paclitaxel plus ramucirumab, and palliative care—irinotecan alone appears to be the most cost-effective. Both paclitaxel alone and the combination of paclitaxel and ramucirumab were not cost-effective with ICER values being $86,815/QALY and $1,056,125/QALY, respectively, more than $50,000/QALY (36). Consistently, nivolumab does not achieve cost-effectiveness compared to placebo for chemotherapy-refractory advanced GC in the current healthcare environment in China (38). Generally, the cost of PD-1 inhibitors is higher in China than in conventional chemotherapy. Based on previous studies and our results, it is suggested that nivolumab plus chemotherapy may not be cost-effective compared to chemotherapy in both first-line and second-line treatment of advanced GC/GEJC/esophageal adenocarcinoma from the perspective of the Chinese healthcare system. As far as we know, since the official establishment of the National Healthcare Security Administration (NHSA) in May 2018, there have been several rounds of negotiations with pharmaceutical companies on the price of cancer drugs, aiming to relieve the medical burden of cancer patients through national strategic procurement (24, 39). At present, the NHSA of China is making great efforts for the successful entry of nivolumab into the negotiation list for the first-line treatment of advanced GC/GEJC/esophageal adenocarcinoma.

Our study has some limitations. First, the model based on the clinical trial and the use of a two-parameter Weibull survival model to extrapolate the long-term PFS and OS beyond the experimental observation time may not accurately reflect the disease course in the real world. Future studies are expected to confirm the cost-effectiveness of nivolumab plus chemotherapy vs. chemotherapy when the clinical data are mature. Second, the HRQoL data for patients were unavailable in the CheckMate 649 trial, and health state utilities used in our study were derived from published literature, which might lead to bias in the model outcomes. However, the result of the sensitivity analysis found that varying the health state utilities in the sensitivity analysis did not substantially change our results. Third, we only considered the most common grade 3/4 SAEs in the model. We hypothesized that low-probability adverse events would not change the final conclusions of the study, and the sensitivity analysis showed that the result was not sensitive to SAE-related parameters. Fourth, according to the guidelines, we assumed all patients subsequently received paclitaxel as salvage chemotherapy, which may not reflect the current Chinese clinical practice situation precisely because patients might choose different treatment options upon further progression. Finally, due to the strict eligible conditions of clinical trials and the unbalanced economic development in various regions of China, the applicability of this study may be limited.

## Conclusion

In conclusion, nivolumab plus chemotherapy is unlikely to be considered cost-effective compared with chemotherapy alone in the first-line therapy for advanced or metastatic PD-L1 CPS ≥5 GC/GEJC/esophageal adenocarcinoma from the perspective of the Chinese healthcare system. However, if the cost is reduced by 90%, nivolumab may be a cost-effective and effective treatment option. Our results may be helpful to provide guidance for GC/GEJC/esophageal adenocarcinoma treatment decisions by physicians and healthcare requests in China.

## Data Availability Statement

The original contributions presented in the study are included in the article/supplementary material. Further inquiries can be directed to the corresponding author.

## Ethics Statement

This article does not contain any studies with human participants or animals performed by any of the authors.

## Author Contributions

YS and QZ made a significant contribution to the work in the conception, study design, execution, acquisition of data, analysis, and interpretation. All authors took part in drafting, revising, or critically reviewing the article and gave final approval of the version to be published.

## Funding

This work was supported by the National Natural Science Foundation of China (No. 82104476).

## Conflict of Interest

The authors declare that the research was conducted in the absence of any commercial or financial relationships that could be construed as a potential conflict of interest.

## Publisher’s Note

All claims expressed in this article are solely those of the authors and do not necessarily represent those of their affiliated organizations, or those of the publisher, the editors and the reviewers. Any product that may be evaluated in this article, or claim that may be made by its manufacturer, is not guaranteed or endorsed by the publisher.
